# *In silico* study of the dynamics of solid food particles in the stomach during gastric digestion

**DOI:** 10.1098/rsif.2025.0291

**Published:** 2025-08-20

**Authors:** Sharun Kuhar, Jung Hee Seo, Rajat Mittal

**Affiliations:** ^1^ Department of Mechanical Engineering, Johns Hopkins University, Baltimore, MD, USA

**Keywords:** computational fluid dynamics, solid food digestion, trituration

## Abstract

In recent years, there has been growing interest in computational fluid dynamics models of the gastric phase of the digestion process. While several models address the digestion and emptying of liquid meals, none incorporate large solid food particles. This omission is significant, as a food bolus typically contains solid particles of varying sizes, with those exceeding 1–2 mm unable to pass through the pylorus. The current study integrates large spherical particles into an imaging-based stomach model to examine the action of hydrodynamic and contact forces on these particles. The model captures particle shuttling dynamics and quantifies the forces that drive trituration in a healthy and a hypomotile stomach at different viscosities of the surrounding liquid contents. The results show that the presence of solid foods can reduce the gastric emptying rate of liquids while also significantly influencing the flow inside the antrum. Hypomotile stomachs were ineffective in trapping the solids next to the pylorus, with many food particles never even making it to the terminal antrum, unlike the healthy case. The pressure, shear stresses and contact forces acting on the solid particles were also lower for the hypomotile case.

## 1. Introduction

In the previous decades, dozens of computational fluid dynamics (CFD) models and studies of the gastric phase of digestion have emerged in the literature [1,2]. These *in silico* models can readily quantify flow velocities [3,4], mixing of contents [5,6], distribution of food and enzymes [7,8] and pH distribution across the lumen [9,10]—measurements that are very challenging to carry out *in vivo*. Such models are often employed to mimic stomach motility disorders and surgeries to study their effects on gastric digestion and emptying [2]. However, despite the rapid developments in the field, CFD models of the stomach have not addressed one key element of gastric function—the breakdown of large solid food particles. Indeed, this was highlighted in a review article from 2017 [11], where it was pointed out that in spite of the numerous analytical and numerical models that have been developed, none have attempted to integrate the behaviour of particles immersed in the flow field. This deficit has remained to the current day [1,2,12] and gastric function with the inclusion of large solid food particles has to-date not been achieved in any *in silico* model of the stomach [3–6,8–10,13–23].

**Figure 1.**
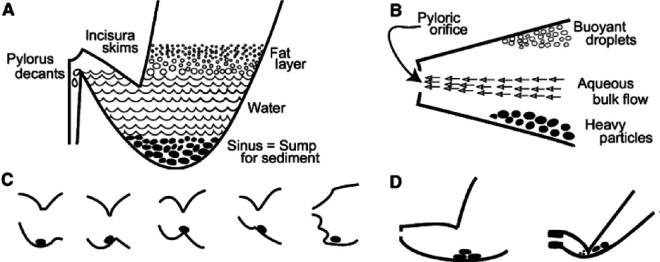
Schematic depicting the implications of settling of solid food particles and the subsequent trituration (used with permission from Schulze [24]). (A) The layering of food contents. (B) Bulk flow carries tiny and isodense particles amidst deflected larger food particles. (C) The *shuttling* motion of particles by the antral contractions propelling portions of the sediments towards the pylorus. (D) Subsequent physical breakdown of those particles.

The current understanding, based on some classical *in vivo* studies [24], highlights the complexities behind the digestion of solid foods. After chewing, the food bolus entering the stomach contains solid particles with sizes ranging from sub-millimetre to 4 mm and larger [25,26]. Subsequently, during the gastric digestion phase, not all of these particles can immediately exit the stomach into the intestines because the pyloric sphincter acts as a sieve that allows only particles smaller than about 2 mm to pass into the duodenum [27,28]. The larger particles must first undergo physical breakdown in the antrum region of the stomach through the combined action of fluid pressure and shear, as well as the action of acidic gastric juice—a phenomenon called trituration [29]. This process continues until the particle size is reduced sufficiently to allow passage through the pylorus. Furthermore, besides the extra processing, large solid food particles also affect the processing of the liquid contents. Since larger particles tend to be heavier, they do not closely follow the motion of the liquid surrounding them due to a larger influence of gravity and inertia. In return, they significantly influence the flow in their vicinity while also altering the motion of other particles. All of these phenomena affect the fluid shear, pressure and mixing inside the stomach, which is essential for the trituration process. Based on several classical experiments over multiple decades, the current understanding notes the critical role of the distal antrum and the pylorus in phase discrimination, sieving and retrograde flow of contents inside the stomach [24] (figure 1). The gastric outlet (pylorus) is located above the level of settled contents, and the pulses of flow carry tiny isodense particles through the pylorus into the descending duodenum. Large and heavy food particles, in contrast, are deflected by the wall contractions. Particles might also be outpaced by the accelerating contractions only to be pushed again by the following contractions. Straining (a process by which the aqueous portion of a mixture passes through and removes bulky material) occurs towards the end of contractions as the pylorus closes. Subsequently, the retrograde flow pushes these bulky particles back into the stomach.

However, existing computational models of the stomach sidestep these complexities by assuming that the contents are purely liquid. A purely liquid meal is, however, a special case because most meal boluses arriving in the stomach include solid components. Even the recommended meal by the Society of Nuclear Medicine and the American Neurogastroenterology and Motility Society for scintigraphy-based diagnosis of emptying rate disorders contains solid foods [30,31]. Despite the liquid meal assumption made by these studies, their results are compared against the scintigraphy studies. The oversimplification arising from assuming purely liquid gastric contents not only fails to address questions about the processing of the solid components but is also erroneous in making predictions about the liquid component of the meals because it does not account for the significant influence of solid particles on the flow and mixing of liquids. Thus, in order to ensure the utility and reliability of model predictions, it is crucial that solid food particles are incorporated into computational models of the stomach.

It has been known for over four decades that the proximal stomach plays a major role in the gastric emptying of liquid meals and the distal stomach plays a major role in that of solid meals [32]. A strong correlation is also reported between antral phasic pressure activity and the rate of emptying of solid food from the stomach [33]. In order to investigate the phenomenon behind this correlation, computational studies incorporating solid foods need to quantify the roles of pressure activity, particle–particle interactions and the hydrodynamic shear stresses acting on the food particles due to antral contractions. However, measurements of these quantities *in vivo* need invasive approaches and are resource intensive. It is also challenging to quantify these forces computationally because introducing large solid food particles into a CFD model introduces new complexities into the system while also increasing the computational cost.

Skamniotis *et al.* [34] simulated the physical breakdown of a bolus of solid food by modelling it as a single large soft-solid using the viscoplastic constitutive law. However, the model did not account for the surrounding fluid and focused only on the solid deformation of the food bolus of 25.6 mm diameter under the action of stomach wall contractions of an idealized stomach geometry. The individual food particles within the bolus were not resolved. In contrast, in this study, we are interested in the forces that lead to the physical breakdown of individual food particles. Despite these limitations, that study was a first-of-its-kind attempt to address one aspect of solid food breakdown *in silico*. Its shortcomings segue into technical challenges, such as the additional computational cost in employing a realistic stomach model and focusing instead on the trituration of individual food particles. In a previous work, we modelled the dissolution of an oral tablet [19], which essentially represents a scenario with a single large solid food particle. Although similar in approach, that prior study did not focus on the forces acting on the solid but rather on the release and transport of the active pharmaceutical ingredient. A high-fidelity model that captures the details of trituration would simulate many such particles, incorporate particle–particle interaction model and discuss the forces acting on the solids.

In this study, we present an imaging-data-based stomach model with multiple large solid food particles too large to pass through the pylorus during gastric digestion. Several features of this configuration, including the forces acting on the particles, particle–particle interactions and the role of terminal antral contraction (TAC) in trituration, are investigated for a healthy stomach and a stomach with antral hypomotility. The study also highlights the significance of including solid foods in computational models and their influence on gastric fluid dynamics.

## 2. Methodology

### 2.1. Stomach model

Similar to our previous study [23], the stomach anatomy used in the current work was generated by modifying publicly available imaging data—Virtual Population Library (VPL) [35]. The original dataset in VPL is representative of an empty or a low-food-volume stomach. To make it resemble a stomach in the postprandial state, the geometry was modified based on publicly available magnetic resonance (MR) images from sources such as the study by Lu *et al.* [36] and the data available on the website of Motilent (London, UK). However, the gastric emptying rate and antral flow characteristics of the new geometry were the same as those in our earlier studies that used a different geometry [8,22], showing that the conclusions are insensitive to the variations in stomach geometry. Also, the study includes only a short segment of the duodenum. Since no duodenal contractions are considered and the focus of the study is on large particles inside the antrum, incorporating a longer duodenum is also not expected to change the results of the study.

**Figure 2.**
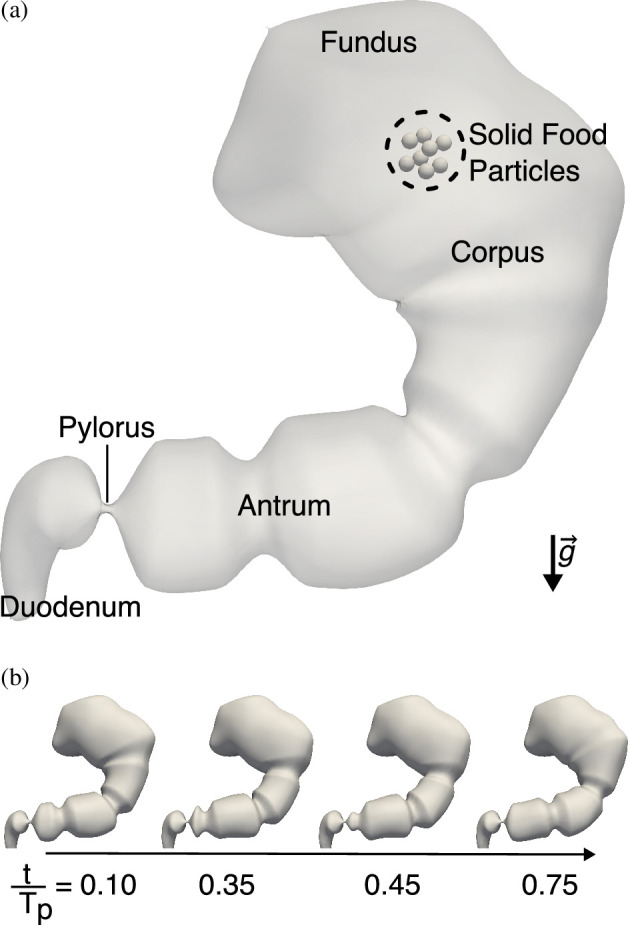
(a) The stomach geometry and the different regions of the stomach. (b) The progression of antral contraction waves over the course of one time period ($T_{p}=20$ s).

The stomach was segmented using the software Mimics (Materialise, Leuven, Belgium), and the shape was exported as a stereolithography file (also known as the STL file format) of the lumen. The mesh was pre-processed (smoothened and re-meshed) using the open-source software MeshLab [37] to improve the mesh quality, and the Vascular Modeling Toolkit [38] was used to obtain the centreline of the stomach. A detailed description of the implementation of stomach motility can be found in our previous work [8,23]. The stomach motility parameters—contraction width, amplitude and frequency—are also the same as in our earlier studies. The final stomach lumen and the wall contractions are shown in figure 2.

### 2.2. Solid food particles model

Solid food particle sizes entering the stomach range from approximately 0.4 to 4 mm [25]. The particles towards the smaller end of this spectrum follow the flow more closely and have a weak influence on the flow field. In contrast, the motion of larger sized particles is governed by a combination of inertia, gravity and flow-induced forces. Furthermore, their presence has a large impact on the surrounding flow. Whereas the smaller particles pass through the pyloric orifice into the intestines, the larger particles undergo trituration in the terminal antrum. In this study, we are interested in studying the forces that induce the trituration of large particles. So, we only consider the particles towards the larger end of the spectrum. All food particles considered in this work are spherical and have a diameter of 4 mm. Since we are interested in solid foods that settle down into the antrum, the density of the solids was chosen to be 1.1 g cm^−3^, which corresponds to meats [39,40] or raw nuts [41,42].

The simulation set-up is inspired by existing *in vivo* studies that studied the digestion of solid foods. A similar configuration was used in an *in vivo* study by Hinder & Kelly [43] where 7−10 mm pieces of digestible and indigestible solids were ingested by four dogs to observe trituration and emptying of solids. We include eight solid spherical particles of 4 mm diameter in our current study. Including more particles in simulations will provide better force statistics, but our choice was driven by two main factors. Firstly, with eight particles, we incorporate a significant volume of solid food in the stomach, and this particle number also provides significant data on particle–particle and particle–antrum interactions. A particle’s experience is primarily influenced by the particles surrounding it. With the current number of particles, we already exceed the volume of contents trapped inside the TAC (denoted by particles lying outside the TAC, as discussed in the results in §3.2). Any additional particles that are introduced would be left out of the TAC collapse. Hence, unless the number of particles increases so much that they fill out the entire antrum, the entrapment of particles and the forces resulting from TAC are not expected to change. Second, the computational expense of the simulations increases rapidly with the introduction of every new solid body because of multiple new pairs of potential contact forces and immersed boundary calculations. With eight particles, each 120 s long simulation costs 28 000–33 000 CPU hours. Striving for a larger number of particles is expected to make the computational cost unreasonable for an initial attempt at modelling solid food digestion.

It is also noteworthy that non-spherical particles are expected to demonstrate some differences in the flow field as compared with spherical particles, albeit locally. We expect the overall trajectory of particles across the stomach (settling down into the sinus and the subsequent recirculation as seen in the results ahead) to be the same as the spherical particles, since it is largely governed by gravity and the push of the antral contraction waves before hydrodynamic retropulsion. The ideal shape assumption captures the overall trajectories of particles while losing shape-sensitive local flow field details. Since this model resolves individual particles using the immersed boundary approach, other shapes can be incorporated in future work.

**Figure 3.**
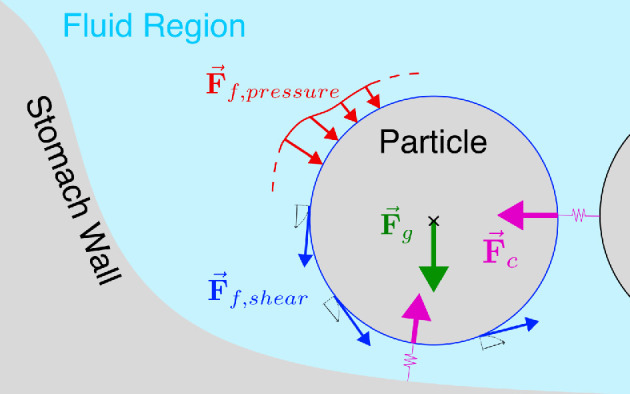
Schematic describing forces acting on a solid food particle. The gravitational force ($F_{g}$) acts on the centre of the particle and the contact forces ($F_{\text{c}}$) act on the point closest to the colliding wall or particle. The fluid forces, i.e. fluid pressure ($F_{\text{f},\text{ pressure}}$) and fluid shear ($F_{\text{f},\text{ shear}}$), act on the entire surface of the particle.

The spherical particles are immersed into the Cartesian mesh and incorporated into the model using the immersed boundary method, like the stomach. The motion of the spherical particles is governed by the equations of motion for the six degrees of freedom of the particle,


(2.1) $$m\frac{dv_{\text{p}}}{dt}=F_{\text{f}}+F_{\text{c}}+mg$$

and


(2.2) $$I\frac{d\omega_{\text{p}}}{dt}=M_{\text{f}}+M_{\text{c}},$$

where $v_{\text{p}}$ and $\omega_{\text{p}}$ are the translational and angular velocities of the sphere, respectively. $F_{\text{f}}$ is the force applied on the sphere by the surrounding fluid, $F_{\text{c}}$ is contact force arising from the spheres colliding with each other or with the stomach walls and g is the acceleration due to gravity. $M_{\text{f}}$ is the moment induced by the surrounding fluid on the sphere, and $M_{\text{c}}$ is that by the contact forces. I is the moment of inertia of the spheres and m is the mass of the sphere. The fluid flow influences the particles’ motion via the hydrodynamic forces and moments arising from pressure and shear stresses. On the flip side, the particles influence the flow field via the no-slip boundary condition at their surface. All forces acting on the particle are described in the schematic shown in figure 3. The gravitational force ($F_{g}$) acts on the centre of the particle, and the contact forces ($F_{\text{c}}$) act on the point closest to the colliding wall or particle. The forces due to the fluid act over the entire particle surface and are composed of a force due to fluid pressure ($F_{\text{f},\text{ pressure}}$) and a force due to fluid shear ($F_{\text{f},\text{ shear}}$).

At t=0, the particles are located in the centre of the upper region of the stomach. They are arranged at the corners of a cube centred at (x,y,z)=(11.5,6.0,9.0) with an inter-particle distance of 0.2 mm so as to avoid contact force springs. This arrangement mimics solid food particles entering the stomach as part of the same food bolus.

The particle–particle and particle–wall collisions are modelled using nonlinear spring-dampers and are described in appendix A. The validation of this particle-resolved approach is carried out by simulating two canonical flow configurations: a sphere falling inside a channel and the draft–kiss–tumble phenomenon of a pair of spheres falling freely in tandem. The results of our model are compared against experimental and other numerical studies in appendix D.

### 2.3. Flow model

The flow inside the stomach is governed by the incompressible Navier–Stokes equations,


(2.3) ∇⋅u=0

and


(2.4) $$\rho \left(\frac{\partial u}{\partial t}+u\cdot \nabla u\right)=-\nabla p+\mu \nabla^{2}u+\rho g,$$

where u is the flow velocity, p is the pressure, ρ and μ are the fluid density and viscosity, respectively. For all cases, ρ=1000 kg m^−3^ is used since most liquid food has a density close to water. Two different viscosities are considered—aqueous or low-viscosity case (μ=1 mPa s) and high-viscosity case (μ=10 mPa s). The details of the numerical methodology are presented in appendix B.

## 3. Results

### 3.1. Particle distribution over time

**Figure 4.**
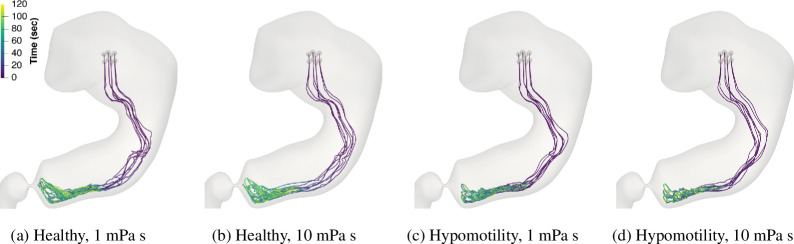
The path of eight food particles over the course of 120 s is shown for the hypomotility and healthy cases at low and high viscosities.

We start by examining the trajectory of particles as they descend due to the force of gravity, followed by subsequent motion under the action of antral contractions. Figure 4 shows the trajectory of all the solid particles as they settle down and their subsequent recirculation within the antrum.

The trajectories show two main features. First is the settling of particles into the antrum. Since the particles are heavier than the fluid medium, this phenomenon is expected and is also well known in the clinical literature [24,44,45]. The second key feature is the subsequent repeated recirculation of these particles within the antrum. We note that the descent takes less than 20 s, and the particles stay within the antrum for the rest of the simulation. Thus, the antral phase of the particles is the most relevant phase for the purposes of trituration.

**Figure 5.**
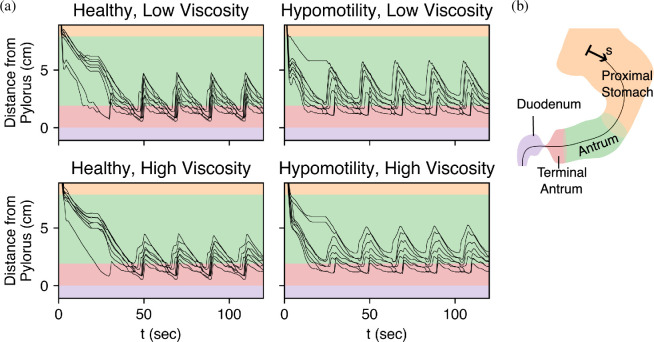
Location of each solid food particle as it descends into the antrum, followed by repeated shuttling under the action of contraction waves. (a) The distance of each particle from the pylorus over time. (b) The colour legend shows different regions of the stomach corresponding to the plots alongside it.

Figure 5 tracks the location of each solid food particle along the centreline of the stomach. All cases exhibit a back-and-forth motion of solid food particles within the antrum. The contractions move these particles closer to the pylorus, the TAC, and the retropulsive jet propels them back into the antrum before the subsequent contraction repeats the cycle. The ‘shuttling’ motion of these particles as they are pushed towards the pylorus, getting trapped between the collapsing stomach walls and then being thrown back repeatedly, is a key mechanism for trituration, the physical breakdown of the food particles into smaller sizes.

The back-and-forth motion is a characteristic feature of solid food digestion inside the stomach, and it has been described in the *in vivo* literature going back to the experiments with canines in 1977 [43]. Kelly [32] describes a cycle of propulsion–grinding–retropulsion of solid food particles, which corresponds to the solids being pushed towards the pylorus, then being grounded as the peristaltic wave passes over them while they are trapped in the distal antrum as they are too large to pass through the pylorus, followed by being propelled backwards towards the proximal stomach, respectively. The understanding has remained consistent over the decades [24,46] and was also described in the schematic earlier (figure 1). Here, for the first time, this phenomenon, which is crucial for trituration, is captured in an *in silico* model.

**Figure 6.**
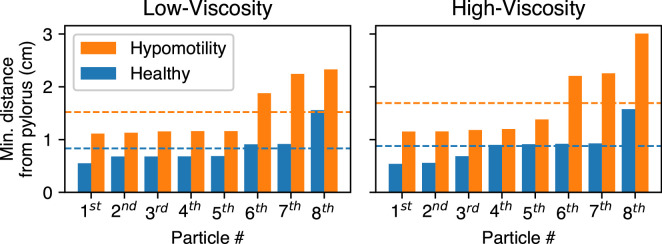
The minimum distance of each food particle in increasing order is plotted for the healthy and the hypomotility case. The dashed line shows the average over the eight particles.

The model also reveals the inefficacy of the hypomotile stomach in achieving this feat. In the low- as well as high-viscosity case, many particles never even make it to the terminal antrum before being slowly pushed back into the antrum, which is in sharp contrast to the healthy case where all the particles get trapped in the terminal antrum and are advected back into the antrum at much higher speeds. To quantify this deficiency, figure 6 shows the closest each particle gets to the pylorus. The average of the minimum distance of food particles from the pylorus in a hypomotile stomach was 83% higher at low viscosity and 93% higher at high viscosity in comparison with the healthy case. We also note that whereas the shuttling motion of the particle in the healthy stomach is relatively insensitive to the fluid viscosity, the food particles are pushed further away from the pylorus in the low-viscosity case, which would diminish their exposure to the high shear near the pylorus. Thus, the healthy stomach exhibits a robustness of trituration to the food viscosity that is not observed for the hypomotile stomach.

### 3.2. Antral contraction wave-driven particle motion

**Figure 7.**
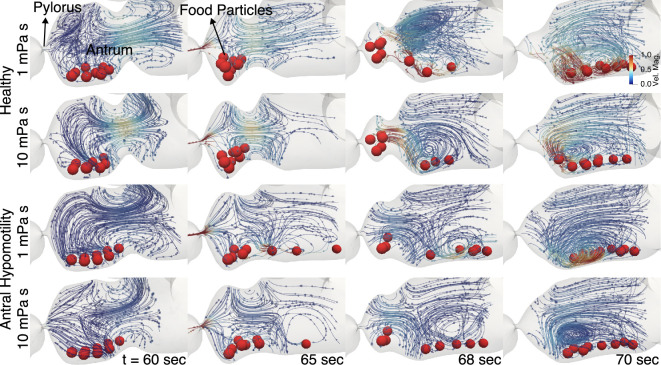
Movement of solid food particles inside the stomach under the action of antral contractions at two different viscosities of surrounding fluid contents for a healthy case and a hypomotility case.

To depict the flow features during the back-and-forth motion of particles, flow visualizations and particle locations for all cases are shown in figure 7 at different instances over the course of one contraction terminating at the pylorus. Streamlines are coloured by velocity magnitudes, and the solid food particles are coloured red.

These visualizations reveal that the healthy stomach can effectively carry and then temporarily trap the solid food particles into the TAC, but this is not the case for the hypomotile stomach. In the healthy cases, the subsequent retropulsion of the particles into the antrum is accompanied by flow activity even in regions away from solid food, while the same is not true for hypomotility cases, where the flow region is largely unaffected away from the particles. Also, in both the healthy and the hypomotility cases, the high flow-velocity regions are more localized as compared with those in low-viscosity cases.

### 3.3. Effect of incorporating solid food particles into the model

**Figure 8.**
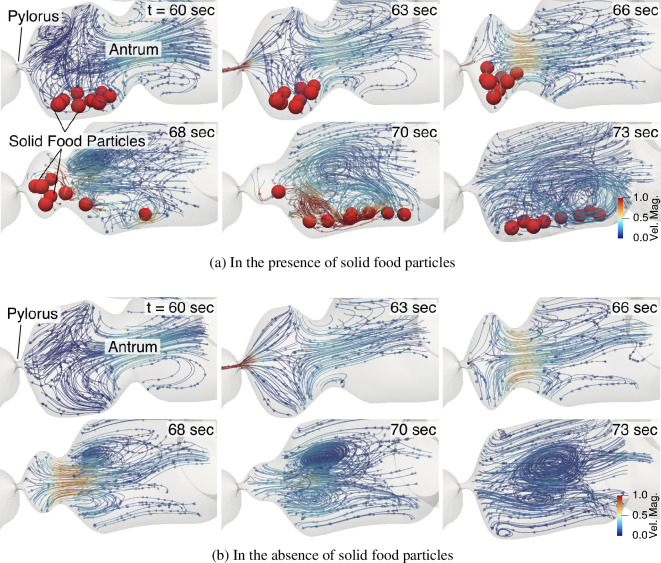
Streamlines showing the highly three-dimensional flow field with and without solid food. The comparison highlights the influence of large solid food particles on the flow inside the antrum.

**Figure 9.**
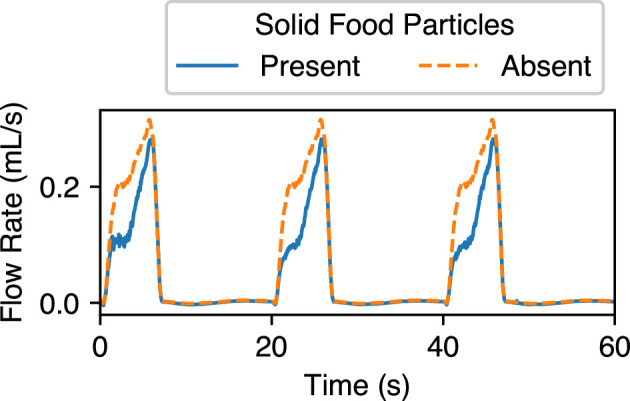
Transpyloric flow rate of the liquid component of the meal in a healthy stomach for two cases: one contains a purely liquid meal, and the other also contains solid food particles.

To examine and quantify the influence of solid foods on the flow, mixing and gastric emptying of the stomach, we also conducted simulations without including the solid particles while keeping all other parameters the same. Figure 8 shows the streamlines of the flow inside the antrum with and without large solid food particles for a healthy stomach with low-viscosity (μ=1 mPa s) contents. It is evident that the size and inertia of these particles significantly influence the flow of contents around them, especially at the time instance when the particles are thrown back into the antrum after TAC (see t=68 and 70 s in figure 8). The influence of the particle is also reflected in the gastric emptying rate of the liquid component of the meal. The transpyloric flux when the stomach contains solid food particles, and when it does not, is compared in figure 9. The differences in the net emptying rates are presented in table 1. Introducing solid foods into the model reduces the emptying rate of liquids by 29% at low viscosity and 5% at high viscosity. The force that pushes the contents through the pylorus also needs to push these particles. As a consequence, a larger volume of fluid can be emptied by the stomach when no solid particles are present. Additionally, when solid foods cannot empty due to their larger sizes, they are trapped before the pylorus and provide further resistance to the transpyloric flow.

**Table 1.** Influence of solid foods on the gastric emptying rate of liquids.

*μ* (mPa s)	emptying rate of liquids (ml min^−1^)	
	no solid food	with solid food
1	4.1	2.9
10	4.1	3.9

### 3.4. Force on particles

The shuttling mechanism shown earlier induces a grinding action on the particles and helps to break down large food particles into smaller ones. The hydrodynamic forces acting on the solid food particles that lead to this breakdown are pressure and shear stress, and these quantities are, therefore, critical to understanding the dynamics of the trituration of solid meals. While there are ways to measure the luminal pressure *in vivo* [47], quantifying the pressure and shear imposed on food particles is not possible. Our current model, however, provides a means to quantify these quantities for the first time.

The mean shear stress on the surface of the particles is calculated using


(3.1) $$\begin{array}{lcr} & <\tau >=\frac{1}{N}\sum_{n=1}^{N}{\bar{\tau }}_{n}\;\text{ and }\;{\bar{\tau }}_{n}=\frac{\int_{n}\tau dA}{\int_{n}dA}, & \end{array}$$

where N=8 is the total number of particles, ${\bar{\tau }}_{n}$ is the mean shear stress on the surface of the *n*th particle and <τ> is the mean over all particles. The mean pressure is calculated in a similar manner using


(3.2) $$\begin{array}{lcr} & <p>=\frac{1}{N}\sum_{n=1}^{N}{\bar{p}}_{n}\;\text{ and }\;{\bar{p}}_{n}=\frac{\int_{n}pdA}{\int_{n}dA}, & \end{array}$$

where ${\bar{p}}_{n}$ is the mean pressure on the surface of the *n*th particle and <p> is the mean over all particles.

**Figure 10.**
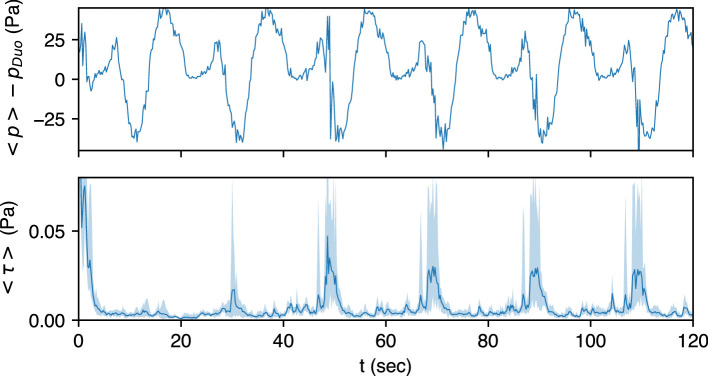
The shear stress and pressure acting on all the particles for the healthy case at low viscosity (μ=1 mPa s). The line shows the average value and the region between the maximum and minimum value of the quantity is represented by the shaded region.

Figure 10 shows the mean over all particles of the shear stress and pressure (i.e. <τ> and <p>) over a 2 min duration plotted as a solid line. The shaded region in the same figure shows the range of those quantities, i.e. the minimum and maximum values of ${\bar{\tau }}_{n}$ and ${\bar{p}}_{n}$. The pressure acting on all particles is found to be quite similar, and this is because pressure is an elliptic variable and has a fairly uniform value in the antrum. In contrast, the shear stress shows significant particle-to-particle variations. This is consistent with the fact that shear stress depends on the local flow features and will, therefore, vary significantly from particle to particle.

**Figure 11.**
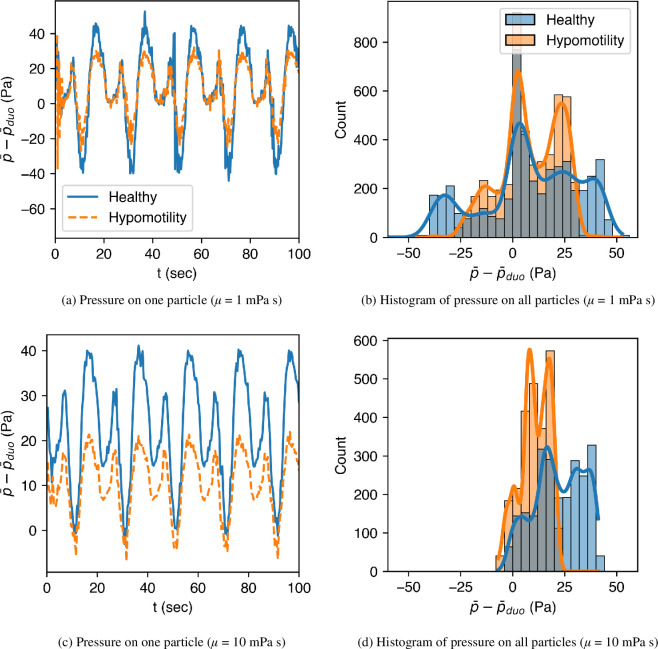
The variation of pressure acting on one solid food particle and the statistics of the pressure acting on all particles shown via a histogram. (*a*) and (*b*) correspond to the case when the surrounding liquid is of viscosity μ=1 mPa s, and (*c*) and (*d*) to that of viscosity μ=10 mPa s.

Figure 11 shows the pressure acting on one of the solid food particles and the histogram of the pressure data for all the particles for the healthy and hypomotile stomachs. The effect of fluid viscosity on the pressure variations is also highlighted in these figures. The figure shows that healthy (normal) motility exposes the particles to larger pressure fluctuations, which is also seen as a wider spread of the histogram. When the gastric contents have a higher viscosity, the fluctuations are smaller, but the average pressure acting on the particles is higher.

The pressure statistics add to our limited understanding of trituration that comes primarily from *in vivo* studies. Apart from revealing the details of particle distribution inside the stomach, the model also highlights that the viscosity of the surrounding medium alters the compressive force acting on the solid foods. Earlier, we saw that both viscosities led to similar trajectories of solid food particles (figure 5) for the healthy stomach. However, the average as well as the range of pressures acting on those particles is quite different for different viscosities.

**Figure 12.**
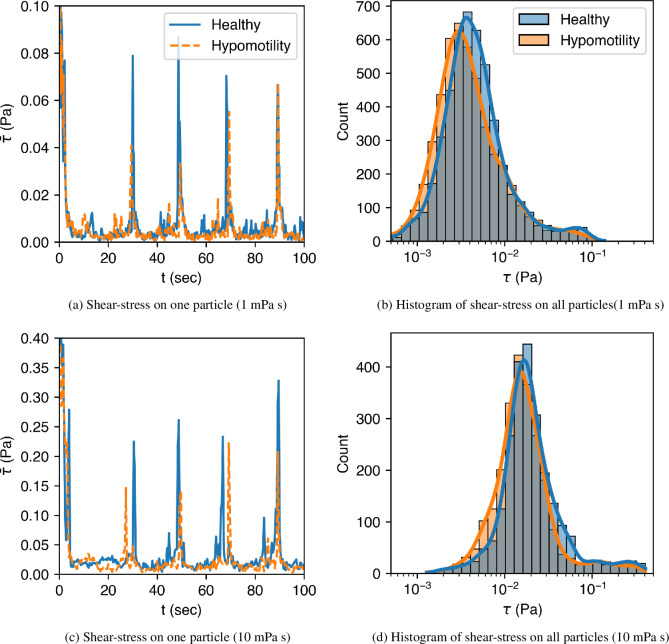
The time variation of the average wall shear stress acting on one solid food particle and the statistics of that on all particles shown via a histogram. (*a*) and (b) correspond to the case when the surrounding liquid is of viscosity μ=1 mPa s, and (c) and (d) to that of viscosity μ=10 mPa s.

The flow of fluid around the particles also leads to shear forces generated on the surface of the particle. Figure 12 shows the shear stresses acting on one of the particles and the histogram of shear stress on all the particles. As expected, higher viscosity leads to much higher shear stresses. The histograms show that lower stresses are more frequent in hypomotility cases as compared with the healthy stomach case. The differences should translate to equivalent differences in the erosion of the solid food particles because the surface shear stresses are found to be highly correlated to particle erosion [48,49].

The summary of statistics associated with hydrodynamic forces is presented in table 2. First, the higher viscosity of gastric contents leads to 83% higher average pressure on the food particles, in the healthy case, because the stomach applies a higher pressure on more viscous contents to achieve the same emptying rate. The rise in average pressure is not seen for the hypomotility case because the particles are not trapped during the TAC, which would have caused the rise in pressure. Second, with increasing viscosity, the standard deviation of pressure drops to almost half of its value for both healthy and hypomotility cases because of the slower movement of food particles when surrounded by a more viscous fluid. The higher momentum diffusivity damps out velocity fluctuations that arise from particle collisions, resulting in lower values of standard deviation and maximum pressure. Lastly, the average shear stress increases by 7.5 times for the healthy case and four times for the hypomotility case at higher viscosity since shear forces directly depend on the fluid viscosity (τ∝∂u/∂n). A similar increase is seen in the standard deviation and the maximum shear stress values.

**Table 2.** Statistics of pressure and shear stress acting on the food particles.

case		$p-p_{duo}$ (Pa)			τ (mPa)		
motility	*μ* (mPa s)	mean	s.d.	max.	mean	s.d.	max.
healthy	1	12	21	53	4	4	79
	10	22	12	41	30	40	343
antral hypomotility	1	10	13	31	5	6	58
	10	11	7	21	20	22	222

**Figure 13.**
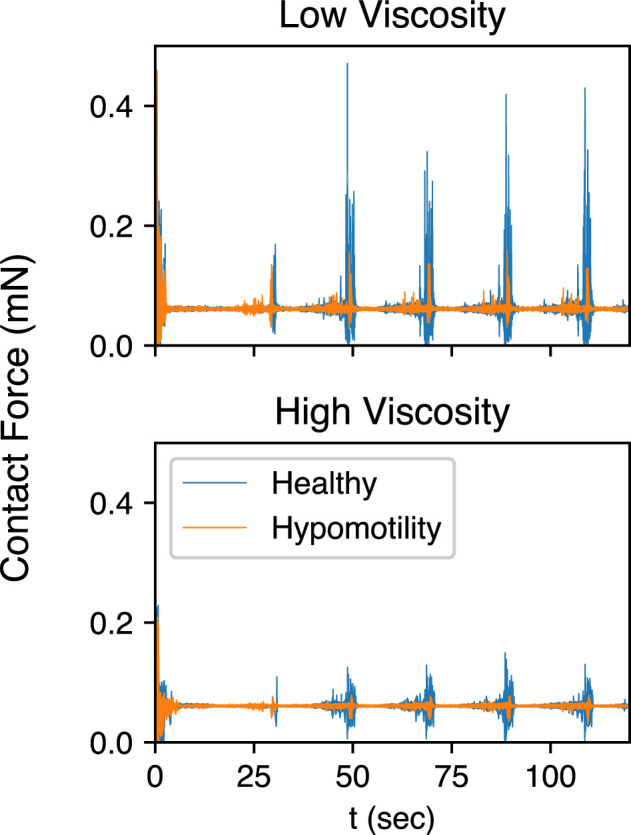
Contact forces acting on all particles are shown for both the healthy and hypomotility cases at low and high viscosities.

Finally, figure 13 shows the magnitude of contact forces ($F_{\text{c}}$ in equation (2.1)) acting on the solid particles for each case. As expected, contact forces show sharp and sudden spikes, which correspond to inter-particle and particle-to-wall collisions. These forces are the highest towards the end of the TAC during which the particles are crushed under the collapsing walls and then ejected back into the antrum. It is noteworthy, however, that contact forces are much weaker for the hypomotility cases due to a weaker TAC. Lower contact forces in hypomotile stomachs would lead to reduced rates of particle erosion and fragmentation of solid food particles, the former being another mechanism for the trituration of solid food TAC. Lower overall erosion and fragmentation would imply a slower gastric emptying rate of solid food particles, which is a defining characteristic of gastroparesis.

In summary, the model allowed us to study the forces contributing to solid food digestion in detail. It also showed the significant influence of motility disorders on trituration. Although useful insights were gleaned using the current model, it has a few limitations. First, the reduction in size of the particles in response to the forces is not incorporated in the modelling procedure. Over the short span of 2 min presented in the current study, however, these effects can be ignored as solid food takes 30−60 min to be broken down into sizes small enough to start emptying through the pylorus [44]. Future studies can try to model these phenomena, but incorporating fracturing and erosion of particles requires models for these complex processes and simulations that extend over much longer durations of the gastric digestion process. Surface erosion can be incorporated into our model by modelling the eroded substance as a passive scalar released from the surface of the particles. The amount of scalar released can be used to drive the rate of change of the particle size. In a future study, it would be interesting to see the transport of this scalar through the stomach and into the duodenum. Another limitation of our current model is that it does not consider other foods alongside the large food particles. The layering of other liquid food contents and the movement of small food particles would also be influenced by the large food particles. Since most food substances have a density close to water and small food particles have a smaller inertia, the impact of these factors on the solid food particles is expected to be insignificant. Finally, although we described a few qualitative comparisons of the findings against *in vivo* reports such as the settling down of particles and the recirculation characteristics, quantitative reports from comparable experiments are missing. Trituration metrics, although available *in vivo*, are only known over very long times, e.g. the half-emptying time of solid meals. Since the study was able to simulate only short durations (120 s), those metrics could also not be used for validation.

Despite its limitations, our model provides multifaceted translational significance. To clinicians, it provides the first of its kind imaging data-based tool that can reveal the dynamics of solid food breakdown, which is a real challenge in gastroparetic patients. The forces experienced by particles can assist in developing finer dietary guidelines for patients while also assisting in surgery planning. To nutritionists and the food industry, it can serve as a high-fidelity tool to design functional foods for specific nutrition and therapeutic needs. Finally, most oral drug delivery assessment tools (such as the United States Pharmacopeia (USP) apparatus or other *in silico* models) only model drug dissolution in a purely liquid environment. Models such as ours can shed light on the effect of other coexisting solid food particles (which is a much more realistic scenario) on the trajectory and rate of dissolution of orally ingested tablets.

## 4. Conclusions

In this study, we develop an imaging data-based computational fluid dynamics model of the stomach, which contains large solid food particles. The goal of the study was to highlight the importance of incorporating solid foods into such models, quantify the relative contributions of forces acting on these particles that bring about their physical breakdown and understand how these forces change when a patient suffers from gastric hypomotility.

The results captured the key characteristics of solid food digestion—settling of solid contents in the antrum and the repeated back-and-forth motion inside the antrum commonly described as a cycle of propulsion–grinding–retropulsion in the literature [32]. In addition, the model showed the effect of the viscosity of the surrounding contents and stomach motility disorders on these phenomena. Where viscosity had little impact on particle trajectories, weaker contractions of the stomach walls significantly affected the stomach’s ability to trap the contents in the terminal antrum before the retropulsion. Unlike the healthy case, many food particles could never even make it to the terminal antrum in the case of hypomotility.

Finally, the model also showed the hydrodynamic and contact forces acting on these food particles and their sensitivity towards the viscosity of surrounding contents and motility disorders. A higher viscosity led to an overall increase in the pressure acting on the solids as well as the surface shear stresses. A stomach with hypomotility, however, also showed much smaller peaks in contact forces, which is expected to result in poor fracturing of solid foods.

## Figures and Tables

**Figure 14.**
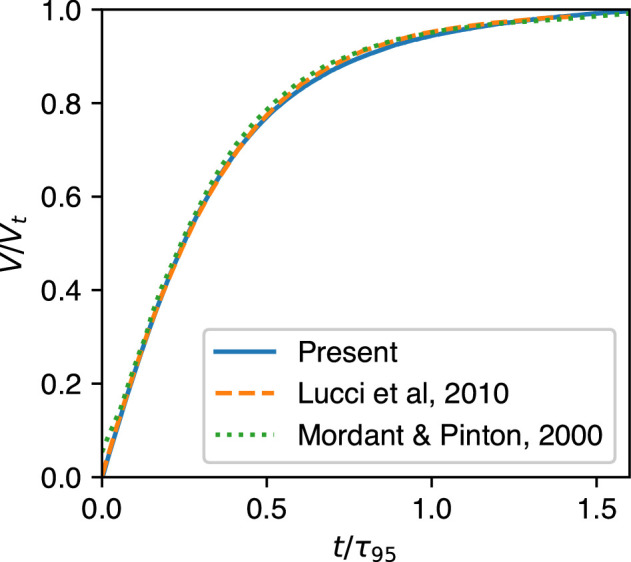
Comparison of normalized velocity against time of a settling sphere.

**Figure 15.**
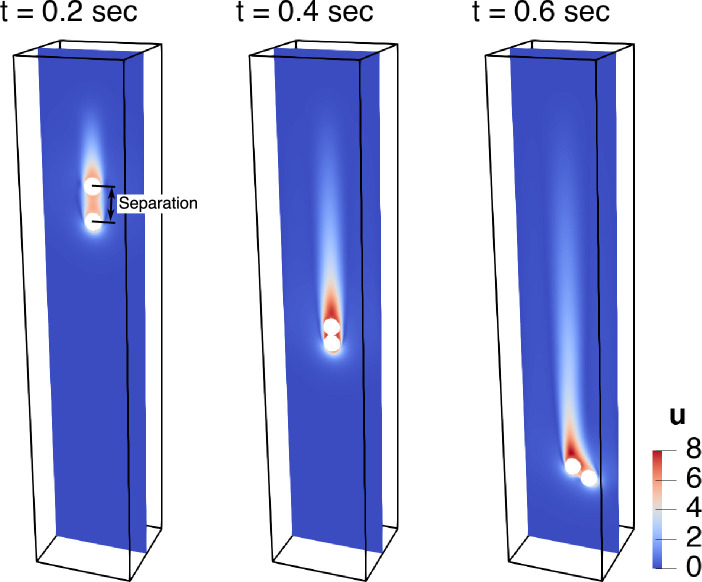
Draft, kiss and tumble.

**Figure 16.**
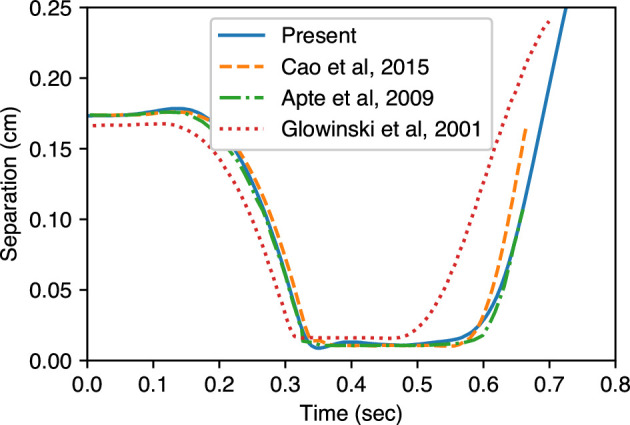
Separation between the two spheres of the same density demonstrating drafting, kissing and tumbling phenomenon.

## Data Availability

The data from this study can be accessed from the github repository: [50].

## Appendix A. Forces on particles

The equations (2.1) and (2.2) describe the translational and angular motion of the spherical particles. The hydrodynamic forces and moments on those particles are given by

(A 1)
$$F_{\text{f}}=\int_{\partial \Omega_{p}}\left(-pI+\tau \right)\cdot n\;dS,\;\text{ and }\;M_{\text{f}}=\int_{\partial \Omega_{p}}r\times \left[\left(-pI+\tau \right)\cdot n\right]dS,$$

where $\partial \Omega_{p}$ represents the surface of the particle, p is the pressure, I is the identity tensor, τ is the shear-stress tensor, r is the vector from the centre of the spherical particle to a point on the particle surface and n is a unit vector normal to the surface at that point. The contact forces and moments on the particles are given by

(A 2)
$$F_{\text{c}}=\int_{\partial \Omega_{p}}f_{\text{c}}\;dS,\;\text{ and }\;M_{\text{c}}=\int_{\partial \Omega_{p}}r\times f_{\text{c}}\;dS,\;$$

where $f_{\text{c}}$ is the contact stress vector at a point on the particle surface due to other nearby bodies, which could be other particle(s) and/or the stomach walls. We use a nonlinear spring model to describe the contact stress vector [18],

(A 3)
$$f_{c}=\sum_{i}f_{\text{c},\text{i}}\;\text{ and }\;f_{\text{c},\text{i}}=\left\{ \begin{array}{ll} -\left(k_{\max}+k_{d}\left(v_{p}-v_{i}\right)\cdot {\hat{r}}_{pi}\right)e^{{\left(-\delta_{i}/\delta_{\min}\right)}^{4}}{\hat{r}}_{pi}, & \text{ if }n\cdot \hat{r}<0\\ 0, & \text{ otherwise } \end{array}\right..$$

Here $v_{p}$ is the velocity of the point on the surface of the particle, $v_{i}$ is the velocity of the nearest point on the surface of the *i*th neighbouring body (with which the particle is in contact, could be another particle or the stomach walls), ${\hat{r}}_{pi}=(r_{i}-r_{p})/|r_{i}-r_{p}|$ is a unit vector from a point on the particle surface to the closest point on the surface of the *i*th neighbouring body and $\delta_{i}=|r_{i}-r_{p}|$ is the distance between those two points. The model has three parameters: $k_{\text{m}\text{a}\text{x}}$, the spring constant; $k_{\text{d}}$, the damping constant and $\delta_{\text{m}\text{i}\text{n}}$, the parameter that controls the range of contact stress. $\delta_{\text{min}}$ is typically set to three grid points, which equals 1.5 mm in our case. Based on a scaling analysis, the maximum contact stress that satisfies the no-penetration condition is used to obtain $k_{\text{m}\text{a}\text{x}}$,

(A 4)
$$k_{\text{m}\text{a}\text{x}}=C_{k}|g|D\;\;\Delta \rho ,$$

where D is the diameter of the particle, $\Delta \rho =\rho_{p}-\rho_{f}$ is the difference between the density of the particle and the fluid, and $C_{k}$ is a constant and is set to 2.5 to ensure no penetration during the fastest collision in the problem, which occurs when particles fall from the proximal stomach onto the stomach walls. The damping constant suppresses non-physical rebounds after the contact using a relaxation time scale,

(A 5)
$$k_{\text{d}}=\frac{\rho_{p}D}{t_{R}},\;\text{ and }\;t_{r}=C_{\text{d}}\Delta t,$$

where $C_{\text{d}}$ is the ratio of relaxation time scale and the simulation time-step size and is set to 5 in our model.

## Appendix B. Numerical methodology

The stomach geometry is meshed with 31 648 triangular elements with triangular edges of approximately 1 mm. Similarly, each solid particle is imported into the solver as a triangulated mesh with 192 elements. The meshed geometries are immersed in a Cartesian grid of size 15×10×13 ${\text{cm}}^{3}$, which is discretized by 300×200×260 grid points. The resolution used was based on a grid independence described in an earlier study [8]. The 0.5 mm grid resolution provides eight grid points across the particle diameter.

The kinematics of the stomach walls are specified as per the contraction parameters described earlier. An in-house finite-difference-based Navier–Stokes solver is used to solve the flow field in response to the moving stomach walls, and the boundary conditions are implemented using the immersed boundary method [51]. The equations are advanced in time using a fraction-step-based algorithm [52]. The spatial derivatives are discretized using a second-order central differencing scheme. The diffusion term is integrated in time using the second-order accurate Crank–Nicolson scheme, and the advection term is updated iteratively. The pressure-Poisson equation is solved iteratively using the stabilized biconjugate gradient (BICG) algorithm [53].

A no-slip boundary condition was prescribed on the stomach walls. A zero-gradient outflow boundary condition was prescribed at the duodenal outflow. Finally, the boundary at the fundus mimics the accommodative function of the function (see Kuhar *et al*. [23] for a detailed description). Inspired by the physiological function of the fundus, this boundary condition applies pressure on gastric contents and drives the gastric emptying of the liquid components. The fundic pressure is adjusted to achieve the desired emptying rate. *In vivo* studies have shown that low-calorie liquid meals empty at the rate of 4.1 ml min^−1^ and are largely insensitive to changes in meal viscosity [54,55]. In accordance, we specified a fundic pressure of 0.1 and 0.2 mm Hg for the low- and high-viscosity cases, respectively, to achieve the same liquid emptying rate.

## Appendix C. Comparison against *in vivo* data

For the case where the stomach contains a purely liquid meal, we have compared the gastric emptying rate of our model with *in vivo* measurements in table 3. The overall rate at which the liquid contents of the stomach empty into the intestines is primarily determined by its caloric density and is relatively insensitive towards variations in meal viscosity [55]. Only two studies report this rate of emptying for a low-viscosity caloric liquid meal. It must be noted that the caloric density of the meal in the present study refers to that of the meal used in the study whose motility parameters are used to inform our model kinematics [3,4].

**Table 3.** Comparison of the gastric emptying rate.

study	viscosity (mPa s)	caloric density (kcal ml^−1^)	emptying rate (m min^−1^)
present	1	0.73	4.09
present	10	0.73	4.15
Brener *et al*. [54]	‘glucose solution’	0.5	4.26
Marciani *et al*. [55]	60	0.64	4.1

We also compared the transpyloric pressure gradient observed in our model with a similar measurement performed in a study by Indireshkumar *et al.* [56] where they used endoscopic manometry to determine the pressure difference across the pylorus using two ports of the manometer separated by 2 cm with one lying in the stomach and the other in the duodenum. Using the same approach, the trans pyloric pressure gradient predicted by the model at different viscosities is shown in table 4. The results at higher viscosities match quite well against the saline solution used in the *in vivo* study.

**Table 4.** Comparison of the transpyloric pressure gradient.

study	viscosity	Δp/L (mm Hg cm^−1^)
present	*μ* =1 mPa s	0.05
	*μ* =10 mPa s	0.09
	*μ* =50 mPa s	0.25
Indireshkumar *et al*. [56]	saline solution of unknown viscosity	0.3±0.2

We also found that the model’s predictions for the gastric emptying rate in disorders and surgeries also match *in vivo* observations. In our previous study [22], gastroparesis patients can have their emptying rates reduced to 44% of healthy individuals, depending on the degree of hypomotility and gastric tone. Urbain *et al.* [57] observed emptying rates drop up to 47% in controls, where the half-emptying times were 62±11 min as compared with 132±17 min in controls. In our study [22], we also quantified the increase in emptying rates after pyloroplasty and found that depending on the post-operative orifice diameter and the gastric tone of the subject, the emptying rates can even be doubled. Similar outcomes are also found in the literature for subjects undergoing pyloroplasty, where the half-emptying time was found to drop from 242.7 to 106.5 min in one study [58] and from 320 to 112 min in another [59], both demonstrating an almost doubled rate of gastric emptying after the surgery. Due to the limited availability of comparable *in vivo* data (such as velocity profiles, mixing efficiency, spatial distribution of enzymes and ingested food), it is currently not possible to report more specific comparisons of the model.

## Appendix D. Validation of the resolved particle model

To validate the numerical solver, we simulate two canonical cases: a solid sphere settling under gravity in a fluid at rest and a pair of in-line spheres of the same density also falling freely in a quiescent fluid under gravity.

### D.1. Settling sphere

The set-up of the problem is the same as the experiment conducted by Mordant & Pinton [60]. A small steel sphere ball ($d_{p}=0.8$ mm, $\rho_{p}$= 7710 kg m^−3^) was released in water ($\rho_{f}=1000$ kg m^−3^, $\nu =9\times 10^{-7}$${\text{m}}^{2}$ s^−1^). The simulation domain was sized $L_{x}\times L_{y}\times L_{z}=1\times 1\times 10$. Under the action of gravity, the sphere accelerates, and the increasing velocity leads to an increasing drag force on the sphere. Eventually, the drag force becomes equal to the gravitational force, and the sphere reaches its settling velocity. Comparison with the literature of the particle’s velocity over this duration is shown in figure 14, and the values of the terminal velocity and the time it takes for the velocity to reach 95% of its terminal velocity are compared in table 5.

**Table 5.** Comparison of terminal velocity and $\tau_{95}$.

study	$V_{t}$ (cm s^−1^)	$\tau_{95}$ (ms)
present	32.8	101.4
Lucci *et al*. [61]	31.6	108.0
Mordant & Pinton [60]	30.9	106.4

### D.2. Drafting, kissing and tumbling

To further evaluate the accuracy of our model, the interactions between the wakes of two identical particles were released with a certain initial separation. Two spheres of diameter 1/6 cm are placed 0.34 cm apart along the axis of a vertical channel. The sphere density is 1.14 g cm^−3^, and the surrounding fluid’s properties are those of water (ρ=1.0 g cm^−3^ and *μ* =0.01 g cm^−1^ s^−1^). More details about the exact same flow configuration can be found in earlier studies that also used it to test their particle-resolved numerical methodologies [62–64].

Figure 15 shows the particle positions at different times, with a cross section through the domain showing contours of velocity magnitudes. In the beginning, the trailing particle lies in the wake of the leading particle and experiences lower drag. Consequently, the trailing particle falls faster and catches up to the leading particle, but when it touches the leading particle, the flow configuration is not stable, and the particles tumble. The phenomenon is known as ‘drafting, kissing and tumbling’ for the three phases described and shown in figure 15. Quantitatively, the separation between the spheres is compared in figure 16, showing that our results are in agreement with existing studies in the literature.

## References

[1] Palmada N, Hosseini S, Avci R, Cater JE, Suresh V, Cheng LK. 2023 A systematic review of computational fluid dynamics models in the stomach and small intestine. Appl. Sci. **13**, 6092. (10.3390/app13106092)

[2] Kuhar S, Mittal R. 2024 Computational models of the fluid mechanics of the stomach. J. Indian Inst. Sci. **104**, 65–76. (10.1007/s41745-024-00421-z)

[3] Pal A, Indireshkumar K, Schwizer W, Abrahamsson B, Fried M, Brasseur JG. 2004 Gastric flow and mixing studied using computer simulation. Proc. R. Soc. Lond. B **271**, 2587–2594. (10.1098/rspb.2004.2886) PMC1691895 15615685

[4] Ferrua MJ, Singh RP. 2010 Modeling the fluid dynamics in a human stomach to gain insight of food digestion. J. Food Sci. **75**, R151–62. (10.1111/j.1750-3841.2010.01748.x) 21535567 PMC2992692

[5] Ishida S, Miyagawa T, O’Grady G, Cheng LK, Imai Y. 2019 Quantification of gastric emptying caused by impaired coordination of pyloric closure with antral contraction: a simulation study. J. R. Soc. Interface **16**, 20190266. (10.1098/rsif.2019.0266) 31387481 PMC6731493

[6] Miyagawa T, Imai Y, Ishida S, Ishikawa T. 2016 Relationship between gastric motility and liquid mixing in the stomach. Am. J. Physiol. Gastrointest. Liver Physiol. **311**, G1114–G1121. (10.1152/ajpgi.00346.2016) 27789458

[7] Trusov PV, Zaitseva NV, Kamaltdinov MR. 2016 A multiphase flow in the antroduodenal portion of the gastrointestinal tract: a mathematical model. Comput. Math. Methods Med. **2016**, 1–18. (10.1155/2016/5164029) PMC4930828 27413393

[8] Kuhar S, Lee JH, Seo JH, Pasricha PJ, Mittal R. 2022 Effect of stomach motility on food hydrolysis and gastric emptying: insight from computational models. Phys. Fluids **34**, 111909. (10.1063/5.0120933) PMC9667910 36407285

[9] Li C, Xiao J, Chen XD, Jin Y. 2021 Mixing and emptying of gastric contents in human-stomach: a numerical study. J. Biomech. **118**, 110293. (10.1016/j.jbiomech.2021.110293) 33588327

[10] Li C, Jin Y. 2023 Digestion of meat proteins in a human-stomach: a CFD simulation study. Innov. Food Sci. Emerg. Technol. **83**, 103252. (10.1016/j.ifset.2022.103252)

[11] Bornhorst GM. 2017 Gastric mixing during food digestion: mechanisms and applications. Annu. Rev. Food Sci. Technol. **8**, 523–542. (10.1146/annurev-food-030216-025802) 28125347

[12] Liu X, Fletcher DF, Bornhorst GM. 2024 A review of the use of numerical analysis in stomach modeling. J. Food Sci. **89**, 3894–3916. (10.1111/1750-3841.17157) 38865250

[13] Imai Y, Kobayashi I, Ishida S, Ishikawa T, Buist M, Yamaguchi T. 2013 Antral recirculation in the stomach during gastric mixing. Am. J. Physiol. Gastrointest. Liver Physiol. **304**, G536–G542. (10.1152/ajpgi.00350.2012) 23275619

[14] Ferrua MJ, Xue Z, Paul Singh R. 2014 On the kinematics and efficiency of advective mixing during gastric digestion – a numerical analysis. J. Biomech. **47**, 3664–3673. (10.1016/j.jbiomech.2014.09.033) 25446267

[15] Berry R *et al*. 2016 Functional physiology of the human terminal antrum defined by high-resolution electrical mapping and computational modeling. Am. J. Physiol. Gastrointest. Liver Physiol. **311**, G895–G902. (10.1152/ajpgi.00255.2016) 27659422 PMC5130547

[16] Harrison SM, Cleary PW, Sinnott MD. 2018 Investigating mixing and emptying for aqueous liquid content from the stomach using a coupled biomechanical-SPH model. Food Funct. **9**, 3202–3219. (10.1039/c7fo01226h) 29775189

[17] Li C, Jin Y. 2021 A CFD model for investigating the dynamics of liquid gastric contents in human-stomach induced by gastric motility. J. Food Eng. **296**, 110461. (10.1016/j.jfoodeng.2020.110461)

[18] Seo JH, Mittal R. 2022 Computational modeling of drug dissolution in the human stomach. Front. Physiol. **12**, 755997. (10.3389/fphys.2021.755997) 35082685 PMC8785969

[19] Lee JH, Kuhar S, Seo JH, Pasricha PJ, Mittal R. 2022 Computational modeling of drug dissolution in the human stomach: effects of posture and gastroparesis on drug bioavailability. Phys. Fluids **34**, 081904. (10.1063/5.0096877) PMC9372820 35971381

[20] Acharya S, Halder S, Kou W, Kahrilas PJ, Pandolfino JE, Patankar NA. 2022 A fully resolved multiphysics model of gastric peristalsis and bolus emptying in the upper gastrointestinal tract. Comput. Biol. Med. **143**, 104948. (10.1016/j.compbiomed.2021.104948) 35091365 PMC9014465

[21] Ebara R, Ishida S, Miyagawa T, Imai Y. 2023 Effects of peristaltic amplitude and frequency on gastric emptying and mixing: a simulation study. J. R. Soc. Interface **20**, 20220780. (10.1098/rsif.2022.0780) 36596453 PMC9810435

[22] Kuhar S, Seo JH, Pasricha PJ, Mittal R. 2024 In silico modelling of the effect of pyloric intervention procedures on gastric flow and emptying in a stomach with gastroparesis. J. R. Soc. Interface **21**, 20230567. (10.1098/rsif.2023.0567) 38263890 PMC10824103

[23] Kuhar S, Seo JH, Pasricha PJ, Camilleri M, Mittal R. 2025 Duodenogastric reflux in health and disease: insights from a computational fluid dynamics model of the stomach. Am. J. Physiol. Gastrointest. Liver Physiol. **328**, G411–G425. (10.1152/ajpgi.00241.2024) 39873302

[24] Schulze K. 2006 Imaging and modelling of digestion in the stomach and the duodenum. Neurogastroenterology Motil. **18**, 172–183. (10.1111/j.1365-2982.2006.00759.x) 16487408

[25] Jalabert-Malbos ML, Mishellany-Dutour A, Woda A, Peyron MA. 2007 Particle size distribution in the food bolus after mastication of natural foods. Food Qual. Prefer. **18**, 803–812. (10.1016/j.foodqual.2007.01.010)

[26] Bornhorst GM, Singh RP. 2012 Bolus formation and disintegration during digestion of food carbohydrates. Compr. Rev. Food Sci. Food Saf. **11**, 101–118. (10.1111/j.1541-4337.2011.00172.x)

[27] Meyer JH. 1980 Gastric emptying of ordinary food: effect of antrum on particle size. Am. J. Physiol. **239**, G133–5. (10.1152/ajpgi.1980.239.3.G133) 7001918

[28] Meyer JH, Dressman J, Fink A, Amidon G. 1985 Effect of size and density on canine gastric emptying of nondigestible solids. Gastroenterology **89**, 805–813. (10.1016/0016-5085(85)90576-1) 4029560

[29] Ramkumar D, Schulze KS. 2005 The pylorus. Neurogastroenterology Motil. **17**, 22–30. (10.1111/j.1365-2982.2005.00664.x) 15836452

[30] Abell TL *et al*. 2008 Consensus recommendations for gastric emptying scintigraphy: a joint report of the American Neurogastroenterology and Motility Society and the Society of Nuclear Medicine. J. Nucl. Med. Technol. **36**, 44–54. (10.2967/jnmt.107.048116) 18287197

[31] Camilleri M, Parkman HP, Shafi MA, Abell TL, Gerson L. 2013 Clinical guideline: management of gastroparesis. Am. J. Gastroenterol. **108**, 18–37. (10.1038/ajg.2012.373) 23147521 PMC3722580

[32] Kelly KA. 1980 Gastric emptying of liquids and solids: roles of proximal and distal stomach. Am. J. Physiol. Gastrointest. Liver Physiol. **239**, G71–G76. (10.1152/ajpgi.1980.239.2.g71) 6996495

[33] Camilleri M, Malagelada JR, Brown ML, Becker G, Zinsmeister AR. 1985 Relation between antral motility and gastric emptying of solids and liquids in humans. Am. J. Physiol. Gastrointest. Liver Physiol. **249**, G580–G585. (10.1152/ajpgi.1985.249.5.g580) 4061646

[34] Skamniotis CG, Edwards CH, Bakalis S, Frost G, Charalambides MN. 2020 Eulerian-Lagrangian finite element modelling of food flow-fracture in the stomach to engineer digestion. Innov. Food Sci. Emerg. Technol. **66**, 102510. (10.1016/j.ifset.2020.102510)

[35] Gosselin MC *et al*. 2014 Development of a new generation of high-resolution anatomical models for medical device evaluation: the virtual population 3.0. Phys. Med. Biol. **59**, 5287–5303. (10.1088/0031-9155/59/18/5287) 25144615

[36] Lu K, Liu Z, Jaffey D, Wo JM, Mosier KM, Cao J, Wang X, Powley TL. 2022 Automatic assessment of human gastric motility and emptying from dynamic 3D magnetic resonance imaging. Neurogastroenterol. Motil. **34**, e14239. (10.1111/nmo.14239) 34431171

[37] CignoniP C, CorsiniM D, GanovelliF R. 2008 Euro graphics Italian chapter conference. (eds V Scarano, RD Chiara, U Erra). Aire-la-Ville, Switzerland: The Eurographics Association. (10.2312/LocalChapterEvents/ItalChap/ItalianChapConf2008/129-136)

[38] Izzo R, Steinman D, Manini S, Antiga L. 2018 The vascular modeling toolkit: a Python library for the analysis of tubular structures in medical images. J. Open Source Softw. **3**, 745. (10.21105/joss.00745)

[39] Rahman MS, Perera CO, Chen XD, Driscoll RH, Potluri PL. 1996 Density, shrinkage and porosity of calamari mantle meat during air drying in a cabinet dryer as a function of water content. J. Food Eng. **30**, 135–145. (10.1016/s0260-8774(96)00013-1)

[40] Kassama LS, Ngadi M. 2016 Shrinkage and density change of de-boned chicken breast during deep-fat frying. Food Nutr. Sci. **07**, 895–905. (10.4236/fns.2016.710089)

[41] Aydin C. 2003 Physical properties of almond nut and kernel. J. Food Eng. **60**, 315–320. (10.1016/s0260-8774(03)00053-0)

[42] Kilanko O *et al*. 2020 Dataset on physical properties of raw and roasted cashew nuts. Data Brief **33**, 106514. (10.1016/j.dib.2020.106514) 33251309 PMC7683228

[43] Hinder RA, Kelly KA. 1977 Canine gastric emptying of solids and liquids. Am. J. Physiol. Endocrinol. Metab. **233**, E335. (10.1152/ajpendo.1977.233.4.e335) 910947

[44] Kong F, Singh RP. 2008 Disintegration of solid foods in human stomach. J. Food Sci. **73**, R67–R80. (10.1111/j.1750-3841.2008.00766.x) 18577009

[45] Mandarino FV, Testoni SGG, Barchi A, Azzolini F, Sinagra E, Pepe G, Chiti A, Danese S. 2023 Imaging in gastroparesis: exploring innovative diagnostic approaches, symptoms, and treatment. Life **13**, 1743. (10.3390/life13081743) 37629600 PMC10455809

[46] Camilleri M. 2015 Gastric motility and gastric emptying. In Yamada’ s textbook of gastroenterology (eds DK Podolsky, M Camilleri, JG Fitz, AN Kalloo, F Shanahan, TC Wang), pp. 348–366, 1st edn. Hoboken, NJ: Wiley. (10.1002/9781118512074.ch20)

[47] Zheng T, BouSaba J, Sannaa W, Eckert DJ, Burton DD, Camilleri M. 2022 Comprehensive characterization of antral and pyloric contractions by high resolution manometry: applied physiology in suspected gastroparesis. Am. J. Physiol. Gastrointest. Liver Physiol. **323**, G255–G264. (10.1152/ajpgi.00119.2022) 35819155 PMC9448275

[48] Abrahamsson B, Pal A, Sjöberg M, Carlsson M, Laurell E, Brasseur JG. 2005 A novel in vitro and numerical analysis of shear-induced drug release from extended-release tablets in the fed stomach. Pharm. Res. **22**, 1215–1226. (10.1007/s11095-005-5272-x) 16078131

[49] Kong F, Singh RP. 2009 Modes of disintegration of solid foods in simulated gastric environment. Food Biophys. **4**, 180–190. (10.1007/s11483-009-9116-9) 20401314 PMC2854610

[50] Kuhar S. 2025 Data for the paper titled: In silico study of the dynamics of solid food particles in the stomach during gastric digestion. Github. https://github.com/sharunkuhar/data_solidfooddigestion10.1098/rsif.2025.029140829641

[51] Mittal R, Dong H, Bozkurttas M, Najjar FM, Vargas A, von Loebbecke A. 2008 A versatile sharp interface immersed boundary method for incompressible flows with complex boundaries. J. Comput. Phys. **227**, 4825–4852. (10.1016/j.jcp.2008.01.028) 20216919 PMC2834215

[52] Chorin AJ. 1969 On the convergence of discrete approximations to the Navier-Stokes equations. Math. Comput. **23**, 341–353. (10.1090/s0025-5718-1969-0242393-5)

[53] Zhu C, Seo JH, Mittal R. 2018 Computational modelling and analysis of haemodynamics in a simple model of aortic stenosis. J. Fluid Mech. **851**, 23–49. (10.1017/jfm.2018.463)

[54] Brener W, Hendrix TR, McHugh PR. 1983 Regulation of the gastric emptying of glucose. Gastroenterology **85**, 76–82. (10.1016/s0016-5085(83)80232-7) 6852464

[55] Marciani L, Gowland PA, Spiller RC, Manoj P, Moore RJ, Young P, Fillery-Travis AJ. 2001 Effect of meal viscosity and nutrients on satiety, intragastric dilution, and emptying assessed by MRI. Am. J. Physiol. Gastrointest. Liver Physiol. **280**, G1227–G1233. (10.1152/ajpgi.2001.280.6.g1227) 11352816

[56] Indireshkumar K *et al*. 2000 Relative contributions of ‘pressure pump’ and ‘peristaltic pump’ to gastric emptying. Am. J. Physiol. Gastrointest. Liver Physiol. **278**, G604–16. (10.1152/ajpgi.2000.278.4.G604) 10762615

[57] Urbain JLC *et al*. 1993 Characterization of gastric antral motility disturbances in diabetes using a scintigraphic technique. J. Nuclear Med. **34**, 576–581. 8455073

[58] Mancini SA, Angelo JL, Peckler Z, Philp FH, Farah KF. 2015 Pyloroplasty for refractory gastroparesis. Am. Surg. **81**, 738–746. (10.1177/000313481508100726) 26140897

[59] Hibbard ML, Dunst CM, Swanström LL. 2011 Laparoscopic and endoscopic pyloroplasty for gastroparesis results in sustained symptom improvement. J. Gastrointest. Surg. **15**, 1513–1519. (10.1007/s11605-011-1607-6) 21720926

[60] Mordant N, Pinton JF. 2000 Velocity measurement of a settling sphere. Eur. Phys. J. B **18**, 343–352. (10.1007/PL00011074)

[61] Lucci F, Ferrante A, Elghobashi S. 2010 Modulation of isotropic turbulence by particles of Taylor length-scale size. J.Fluid Mech. **650**, 5–55. (10.1017/S0022112009994022)

[62] Glowinski R, Pan TW, Hesla TI, Joseph DD, Périaux J. 2001 A fictitious domain approach to the direct numerical simulation of incompressible viscous flow past moving rigid bodies: application to particulate flow. J. Comput. Phys. **169**, 363–426. (10.1006/jcph.2000.6542)

[63] Apte SV, Martin M, Patankar NA. 2009 A numerical method for fully resolved simulation (FRS) of rigid particle–flow interactions in complex flows. J. Comput. Phys. **228**, 2712–2738. (10.1016/j.jcp.2008.11.034)

[64] Cao C, Chen S, Li J, Liu Z, Zha L, Bao S, Zheng C. 2015 Simulating the interactions of two freely settling spherical particles in Newtonian fluid using lattice-Boltzmann method. Appl. Math. Comput. **250**, 533–551. (10.1016/j.amc.2014.11.025)

